# Unusual Local Therapies Used for the Treatment of Low Back Pain and Sciatica: Concepts and Approaches

**DOI:** 10.7759/cureus.17080

**Published:** 2021-08-11

**Authors:** Bilal Khan, Ikram Alam, Usman Haqqani, Sajjad Ullah, Saima Hamayun, Khalid Khanzada, Zohra Bibi

**Affiliations:** 1 Neurosurgery, MTI-Lady Reading Hospital, Peshawar, PAK; 2 Neurosurgery, Government Naseer Ullah Babar Memorial Hospital, Peshawar, PAK; 3 Neurosurgery, Qazi Hussain Ahmed Medical Complex, Nowshehra, PAK; 4 Neurosurgery, MTI-Khyber Teaching Hospital, Peshawar, PAK; 5 Medicine, Khyber Medical College, Peshawar, PAK; 6 Neurosurgery, Ibrahimi Medical Center, Peshawar, PAK; 7 Psychiatry, MTI-Lady Reading Hospital, Peshawar, PAK

**Keywords:** local therapies, alternative therapies, low back pain, sciatica, stabbing the back, stabbing the leg, drawing blood from the legs

## Abstract

Background and objective

Low back pain (LBP) and sciatica are major healthcare issues globally. Since patients may seek various ways to cure their ailments, these conditions are managed not just by physicians, but many other health-related professionals provide alternative treatment options for it as well. We conducted this study to examine a local subset of patients who used stabbing their back and legs as a treatment option for curing LBP and sciatica.

Materials and methods

This cross-sectional study was conducted in the outpatient clinic of the Neurosurgery unit of Government Naseer Ullah Babar Memorial Hospital, Peshawar, Pakistan, from July 2019 to March 2020. Patients who presented to the outpatient department (OPD) with complaints of LBP with or without sciatica, with a history of invasive therapy in the form of stabbing the back or leg, or drawing blood from the veins of the lower limbs, were included. All other patients with LBP seeking neurosurgical advice were excluded from the study. The study was approved by the management of the hospital and informed consent was obtained from the patients before interviewing them. Special permission was taken for publishing the photographs. The demographics and clinical information related to patients, such as age, gender, duration of symptoms, time since the local therapy, particulars of the treatment provider, any relief experienced by the patient, duration of relief, the patient beliefs/notions about the therapy and disease, and education level of the patients, were recorded on a predesigned form after taking informed consent. The study was done on purposive sampling. The data was presented in tables and charts and was analyzed using SPSS Statistics version 20 (IBM, Armonk, NY).

Results

During the study period, more than 8,000 patients visited the neurosurgical OPD, and the majority of them (>70%) sought treatment for LBP and sciatica. Of them, around 130 patients had a history of undergoing some alternative therapy that is not scientifically proven, and it was either in the form of stabbing the back or drawing blood from the veins in the lower limbs. Amongst these patients, almost 80% were males and 20% were females who had undergone this kind of treatment. The age range among the cohort was 25-68 years and the mean age was around 43 years. The duration of symptoms ranged from two months to nine years, and the time since the therapy and patient seeking medical advice ranged from three months to 4.5 years. The treatment had been provided by a local individual who did not hold any medical degree according to the patients in 100% (n=130) of the cases; 67% of patients felt they had experienced some relief from the therapy for a short period, which ranged from three days to one month. About the condition, none of the patients seeking the therapy knew it was nerve-related and were often confused about the term *rugg* (vessel in the native language) but could not differentiate it from the nerve. Of these patients, 76 required surgery while 54 were managed conservatively for LBP and sciatica.

Conclusion

A subset of the local population in our part of the world used stabbing the back and leg as a form of therapy for treating the problems of the lower back and sciatica. This has not been previously reported and has no scientific basis. Also, the majority of the patients were uneducated and had very little knowledge of the disease, and the treatment provider was a non-health-related professional.

## Introduction

Low back pain (LBP) is defined as the pain that typically occurs between the lower rib margins and the buttock creases; it may or may not be accompanied by radiating leg pain. LBP is a common medical problem worldwide, and it affects people of all ages, from those of school-going age to the elderly [1]. According to one study, it is estimated that more than 80% of the population suffers from LBP at some point in their lives [2]. This can be acute or chronic, and patients can experience acute exacerbation with chronic LBP [1]. There are many causes of LBP apart from clear-cut anatomical reasons; it may also arise from the pathologies of the bony spine, spinal cord/nerves, facet joints and muscles, or the disturbance of sagittal balance [2,3]. Many tests and investigations are used to investigate the causes of LBP, and these range from simple X-ray films to CT and MRI scans [4]. The back or the spinal column is a gray area and is the focus of interest among many disciples of medicine. The types of interventions in the spinal column range from injections in the epidural space to microdiskectomies, spinal instrumentations, and corpectomies [5].

Apart from the mainstream therapies, alternative therapies are also used for treating LBP in many parts of the world. These alternative therapies are in the form of acupuncture, stretching, manipulative treatment, natural healing, respiratory control, and meditations [6]. Regional variations do exist in these methods, and there are therapies with no scientific basis and are based on local cultural assumptions and practices [6,7]. We have come across patients with complaints of LBP or sciatica who had undergone alternative treatments such as stabbing their back or leg or withdrawing blood from the lower limbs based on the impression of it being "bad blood". We conducted this study to analyze the various types of such therapies used by these patients, their understanding/notions about it and the disease, the need for any surgery, and if these treatments had provided any help in relieving their symptoms in the interim period.

## Materials and methods

This cross-sectional study was conducted in the outpatient clinic of the Neurosurgery unit of Government Naseer Ullah Babar Memorial Hospital, Peshawar, Pakistan, from July 2019 to March 2020. Patients who presented to the outpatient department (OPD) with complaints of LBP with a history of undergoing some invasive therapy in the form of stabbing the back or leg, or drawing blood from the veins of the lower limb, were included in the study. All other patients with LBP seeking neurosurgical advice were excluded from the study. We obtained approval for the study from the management of the hospital, and informed consent from patients was taken before interviewing them. Special permission was taken with respect to photographs. We recorded the demographic and clinical details of patients, such as age, gender, duration of symptoms, time since the local therapy, particulars of the treatment provider, any relief experienced by the patient, duration of relief, the patient concepts about the therapy and the disease, and education level of the patients, on a predesigned form after taking informed consent. The study was done on purposive sampling. The data was depicted in tables and charts and was analyzed using SPSS Statistics version 20 (IBM, Armonk, NY).

## Results

**Figure 1:**
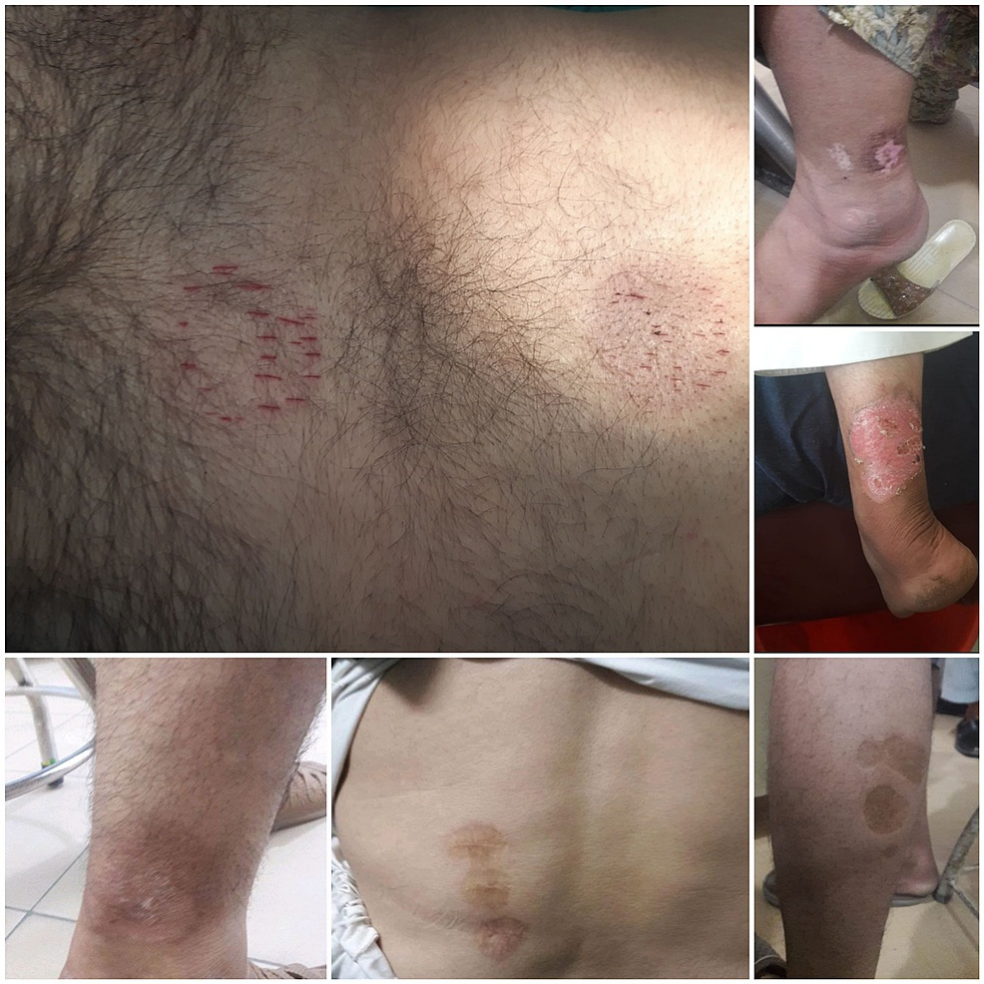
The images show the back and legs of patients who had undergone stabbing as a treatment option for LBP and sciatica LBP: low back pain

During the study period, more than 8,000 patients visited the Neurosurgical OPD, and the majority of them (>70%) were seeking treatment for LBP. Among them, around 130 patients had a history of receiving some kind of alternative therapy like stabbing the back/leg or drawing blood from the veins of lower limbs (Figure 1).

Amongst these patients, almost 80% were males and 20% were females who had undergone this kind of treatment. Their ages ranged from 25 to 68 years and the mean age was around 43 years. The duration of symptoms ranged from two months to nine years, and the time since therapy and patient seeking medical advice ranged from three months to 4.5 years. The treatment was provided by a local individual who did not hold any medical degree according to the patients in 100% (n=130) of cases; 67% of the patients thought they had felt some relief from the therapy for a period, which ranged from three days to one month. About the condition, none of the patients seeking this therapy knew it was nerve-related and were often confused about the term rugg (vessel in the native language) but could not differentiate it from the nerve. Of note, 76 patients required surgery while 54 were managed conservatively for LBP and sciatica (Table 1).

**Table 1:** Demographics and clinical details of patients with LBP and sciatica who used alternative therapies LBP: low back pain; SD: standard deviation

Variables	Values/findings
Age in years	Range: 25-68 (mean ±SD: 43 ±4.5)
Gender, (M/F), %	80/20
Duration of symptoms	2 months-9 years (mean ±SD: 1.3 ±0.6 years)
Time since therapy	3 months-4.5 years (mean ±SD: 1.2 ±0.5 years)
Educational qualification of patients	65% uneducated
Qualification of treatment providers	100% were not health-related
Relief in symptoms (temporary) among patients	67%
Knowledge about the condition (differentiation between nerve and vessel)	100% could not differentiate
Need for surgery	76/130

## Discussion

LBP is a major medical problem worldwide and is one of the top five health-related issues. It can lead to patients being apprehensive and seriously affect their quality of life so that the indirect costs of it are far greater than the direct costs of treatment [8,9]. This persuades many people to not just seek professional medical advice but also undergo alternative forms of therapies in the hope of getting some relief, though some of these modalities may have very little if any scientifically proven efficacy [10]. We have often come across patients who had received alternative therapies such as stabbing their back and legs as a treatment option for sciatica and LBP, a mode of therapy used by the local population but has never been reported in the medical literature, to the best of our knowledge.

As noted, about 8,000 patients attended the Neurosurgical OPD during the study period, and almost 70% of the consultations were regarding LBP and sciatica. Among these consultations, only 130 patients had undergone this form of local therapy, which is relatively a small number. But since they had used it most often for chronic LBP (>3 months' duration), which has a prevalence rate of about 8% [7], it meant that such patients comprised a considerable segment of all patients with chronic LBP. The patients attended our OPD within a period ranging from one month to 4.5 years of undergoing this form of local therapy, and about 67% had experienced some relief of symptoms from this modality of treatment for a short period, which lasted for about a couple of days to a month. While we were unable to learn much about this method as a treatment option for sciatica and LBP, and could not explain the possible interim relief reportedly provided by it, the possible reasons for this short term relief can be attributed to various factors, such as the natural course of LBP and sciatica [about 86-94% of patients with chronic LBP are pain-free at five and 10 years [11,12], and about 60-76% of sciatica resolve spontaneously with time [13,14]; also, the effect of sharp pain experienced by the patient due to the treatment may be really intense, leading them to ignore the chronic pain of LBP itself: conditioned pain modulation (CPM) theory] [15]. Moreover, the pain may also resolve due to the psychosocial effects of the patients' beliefs on the outcomes, as molded by the treatment provider [16].

In our study, 65% of the patients were uneducated, and the treatment provider was a non-health-related professional in almost 100% of cases. This is the result of decades-long practices that have roots in the region and has been inculcated in the minds of the lay public, long before the establishment of the first-ever neurosurgery unit in the area (which was about four decades ago), a region which now harbors a population of more than 35 million. Furthermore, in the initial period, physicians treating LBP and sciatica themselves focused on structural, anatomical, and biomechanical factors (SAB model), with more focus on the invasive nature of treatment [10]. Also, it is possible that the early results of surgeries instilled fear in the minds of people as most of them used to suggest that back surgeries may lead to paraplegia, a belief instilled by the alternative treatment provider rather than the lay public, which may have led them to avoid surgical approach or consultation. Furthermore, these alternative treatment providers often display high confidence in their methods, and thereby exploit the patients' vulnerability to convince them of the benefits of these alternative methods. This is often made possible by the fact that patients are often not well educated enough to understand the basic nature of their disease, and they usually seek the advice of these alternative providers as a first option because of ease of accessibility and low cost. It is evident that the beliefs/behavior of treatment providers and patients have a direct effect on the outcomes [16]. Since almost none of these patients understood the anatomic difference between a nerve and an artery, it presented a formidable challenge to mold their concept and ingrain concrete ideas about the disease, even with concerted efforts and expensive campaigns, as evidenced by the fact that such campaigns have not yielded promising results even in highly educated societies [17].

In most cases, the main impetus for the general public to seek these kinds of alternative treatment is their personal observations, information obtained from fellow laymen, and the experience of their counterparts [10,18]. Such information is easily exchangeable and promulgated at get-togethers like community gatherings, and because the general public is more susceptible to it due to their lack of education, it easily gains acceptability and popularity among them. However, it is essential to invest time and efforts to raise awareness among the general public to help them understand the basics of the disease through ventures involving people who had successfully undergone treatment of LBP and sciatica, and with the help of general physicians. Equally important are mass campaigns about the benefits of modern imaging studies like MRI and CT scans, through platforms such as general public gatherings and social or mainstream media. Such efforts will definitely have some beneficial impact on the public's perceptions in the future.

This was a retrospective study based on patients' memory and their ability to recall their experiences. Many of them were not aware of the use of pain killers during the study period, and it could not be determined if the short-term relief had been totally provided by the treatment. Also, both the treatment seeker and provider did not have sufficient knowledge and qualifications to explain it scientifically.

## Conclusions

LBP and sciatica are major health-related issues, and a major concern involves the indirect costs incurred by loss of work and productive life. Patients seek treatment from not just physicians but also many health-related professionals like physiotherapists, chiropractors, etc. The local population in our part of the world often uses alternative therapies like stabbing the back and leg as a form of therapy for treating the problems of the lower back and sciatica. This has not been previously reported and does not have any scientific basis. The short-term relief reportedly provided by such methods is not due to the treatment effect but a result of many confounding factors. The majority of these patients were uneducated and had very little knowledge about the disease, and the treatment provider was a non-health-related professional. Efforts need to be made to reduce people relying on such practices with the help of general physicians, patients successfully treated with proper methods, and through mass social and mainstream media campaigns.
